# Supplementary material for: Assessment of metrics in next-generation sequencing experiments for use in core-genome multilocus sequence type
Source: PeerJ. 2021 Aug 19;9:e11842. doi: 10.7717/peerj.11842 (PMC8380430; doi:10.7717/peerj.11842)
Supplement: Supplemental Information 1 — Comparison of different read lengths for the assembling running time according to different simulated read coverages among SPAdes, CLC, and SKESA for S. enterica LT2. [file peerj-09-11842-s001.pdf]

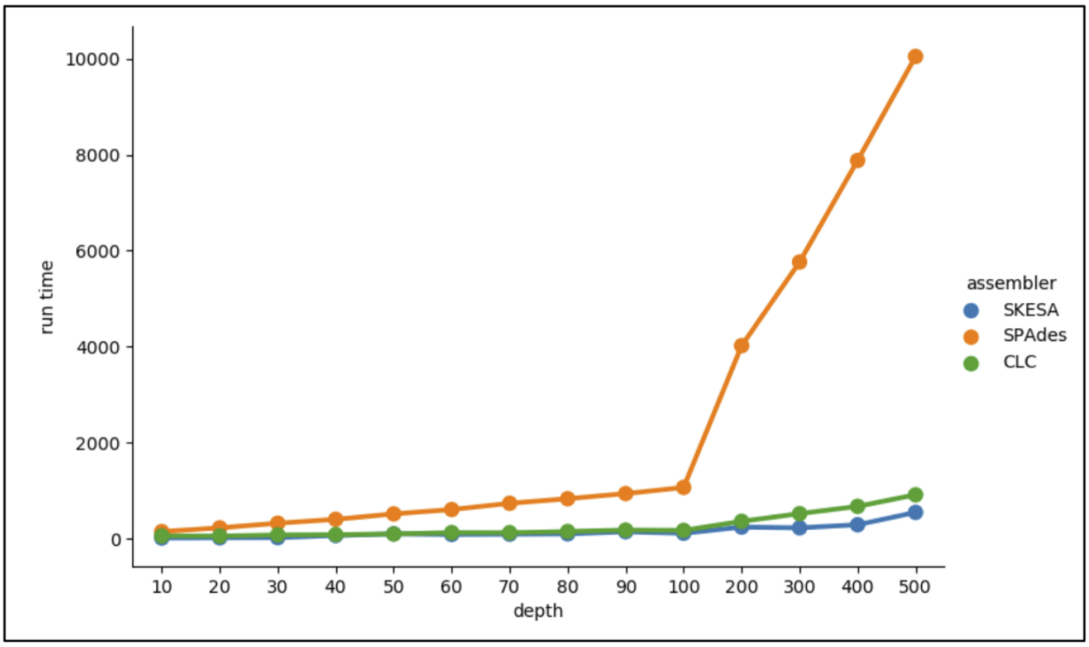

Figure S1. The estimation of the minimum read coverage for the running time of SPAdes, CLC, and SKESA assemblers. Comparison of different read lengths for the assembling running time according to different simulated read coverages among SPAdes, CLC, and SKESA for *S. enterica* LT2.
