# Supplementary material for: Assessment of metrics in next-generation sequencing experiments for use in core-genome multilocus sequence type
Source: PeerJ. 2021 Aug 19;9:e11842. doi: 10.7717/peerj.11842 (PMC8380430; doi:10.7717/peerj.11842)
Supplement: Supplemental Information 2 — The “Per sequence quality scores” and “Sequence Length Distribution” from QC reports of (A) S. enterica (SRR5866640 for 150 bp and SRR6929558 for 250 bp), (B) E. coli (SRR6924239 for 150 bp and SRR3205757 for 250 bp), and (C) L. monocytogenes (SRR3089759 for 150 bp and SRR6347431 for 250 bp) were shown. [file peerj-09-11842-s002.pdf]

(A) *S. enterica*

SRR5866640 (150bp)

Per sequence quality scores | **Sequence Length Distribution**

Read 1

Read 2

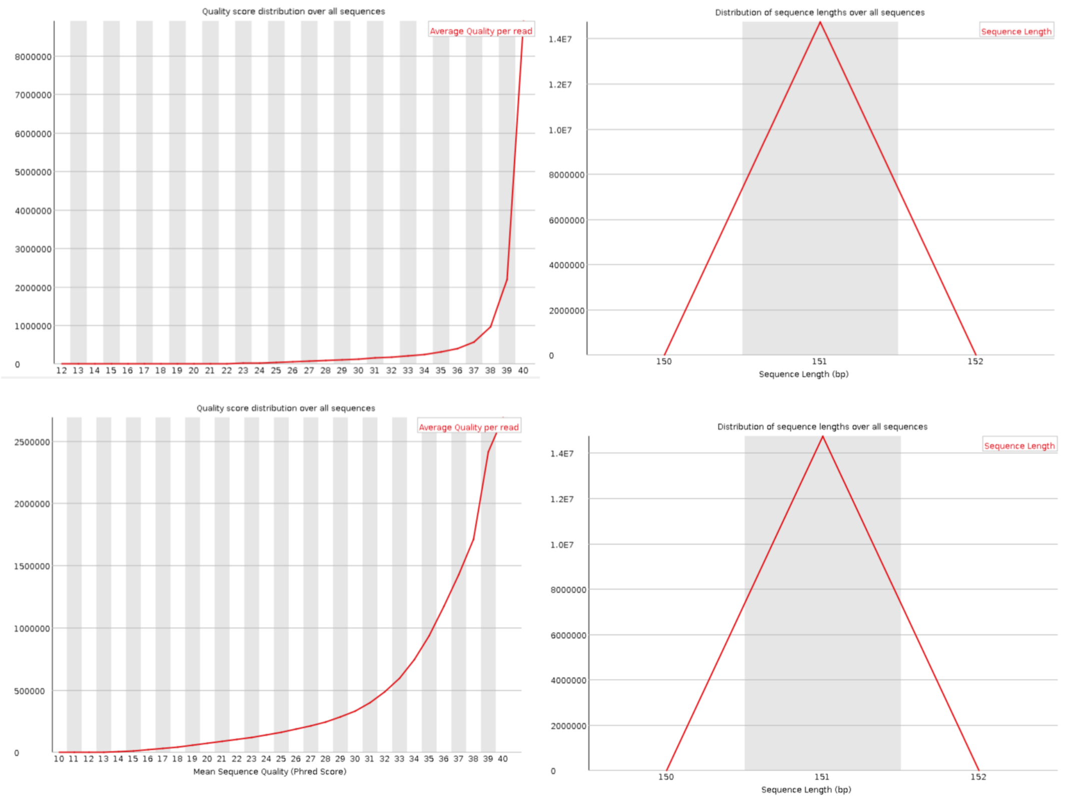

SRR6929558 (250bp)

Read 1

Read 2

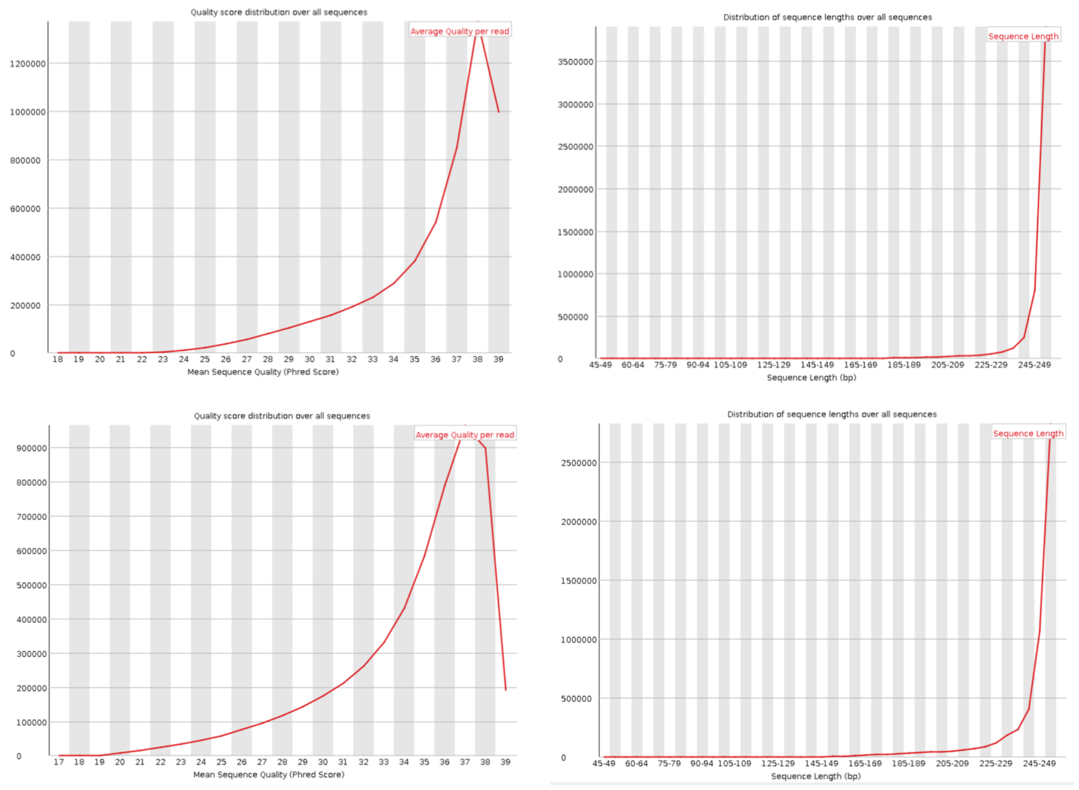

(B) *E. coli*

SRR6924239 (150bp)

Per sequence quality scores | **Sequence Length Distribution**

Read 1

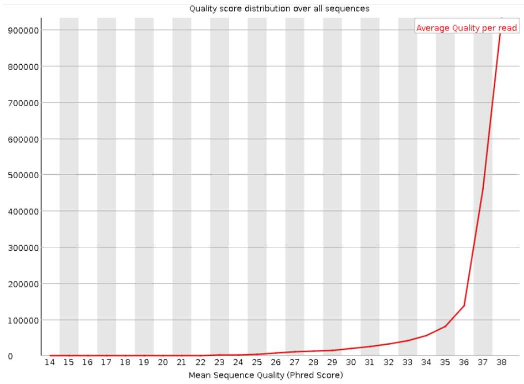

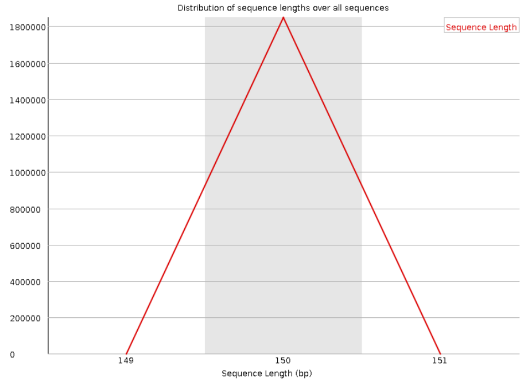

Read 2

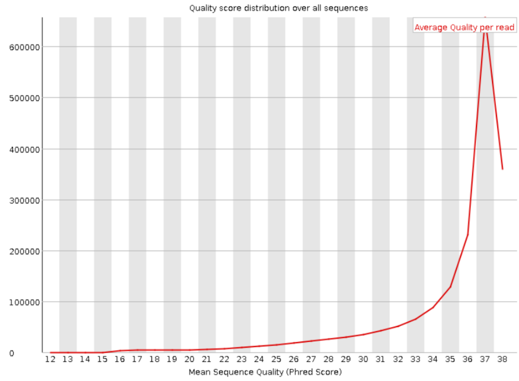

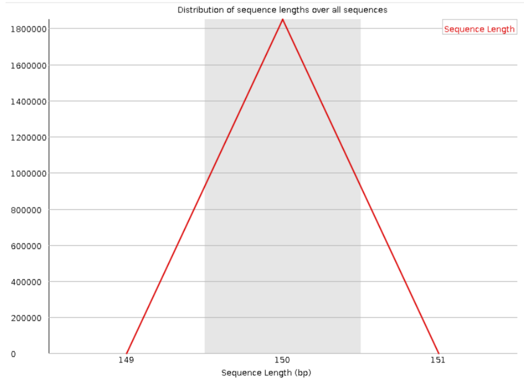

SRR3205757 (250bp)

Read 1

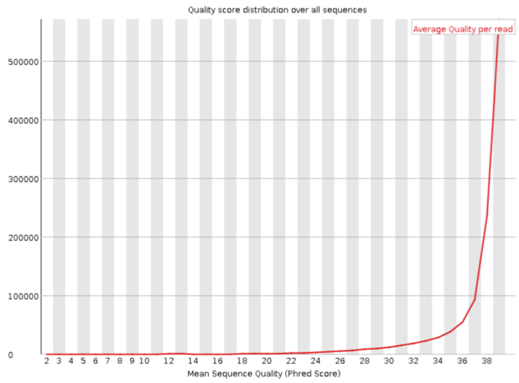

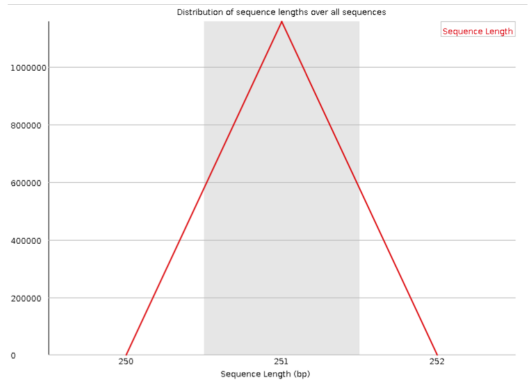

Read 2

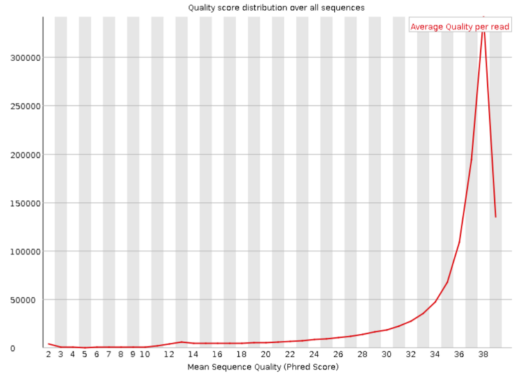

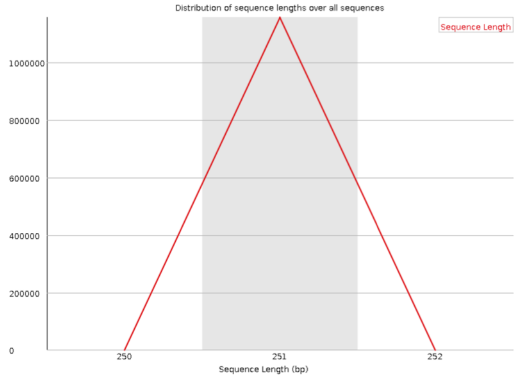

(C) *L. monocytogenes*

SRR3089759 (150bp)

Per sequence quality scores | **Sequence Length Distribution**

Read 1

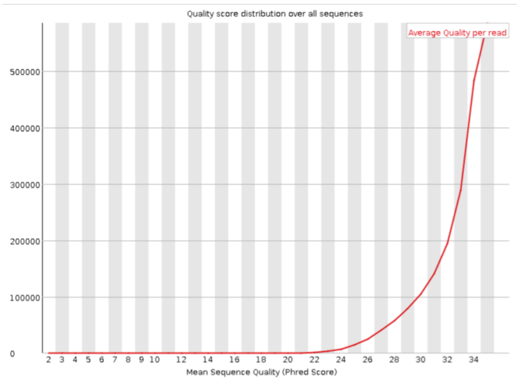

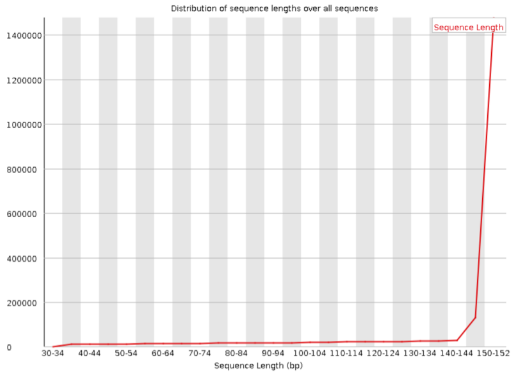

Read 2

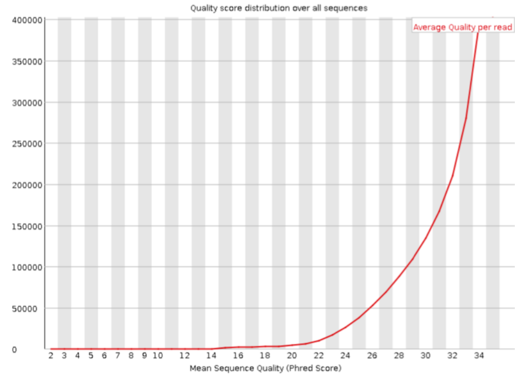

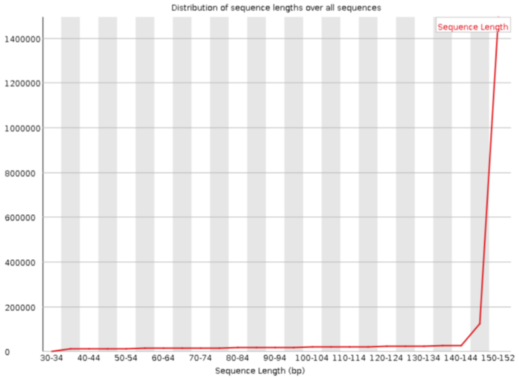

SRR6347431 (250bp)

Read 1

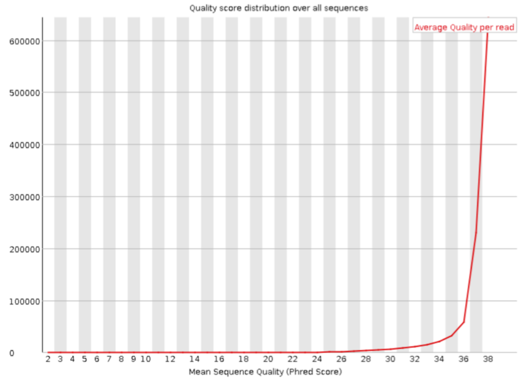

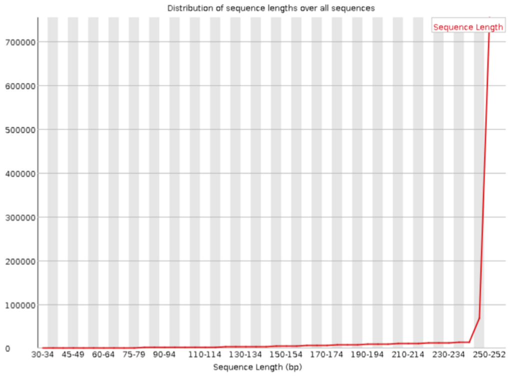

Read 2

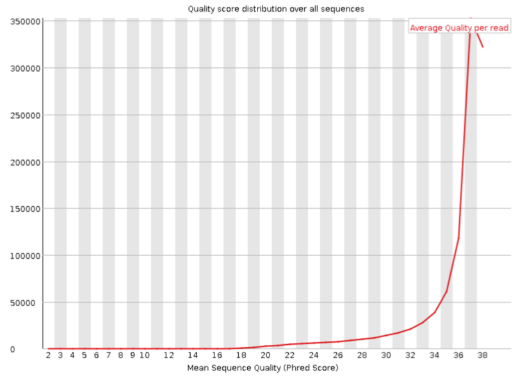

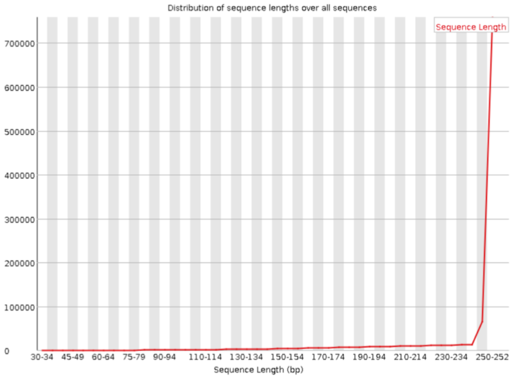

Figure S2. The real sequenced reads QC performed by using FastQC. The “Per sequence quality scores” and “Sequence Length Distribution” from QC reports of (A) *S. enterica* (SRR5866640 for 150 bp and SRR6929558 for 250 bp), (B) *E. coli* (SRR6924239 for 150 bp and SRR3205757 for 250 bp), and (C) *L. monocytogenes* (SRR3089759 for 150 bp and SRR6347431 for 250 bp) were shown.
