# Supplementary material for: Assessment of metrics in next-generation sequencing experiments for use in core-genome multilocus sequence type
Source: PeerJ. 2021 Aug 19;9:e11842. doi: 10.7717/peerj.11842 (PMC8380430; doi:10.7717/peerj.11842)
Supplement: Supplemental Information 4 [file peerj-09-11842-s004.pdf]

Table S2. The annotation of unrecalled core genes at depth 100×.

S. enterica	***E. coli***	***L. monocytogens***
Anaerobic dimethyl sulfoxide reductase chain B	Cellulose biosynthesis protein BcsG	IS3 family transposase ISLmo1
Anaerobic dimethyl sulfoxide reductase chain C	Cysteine--tRNA ligase	hypothetical protein
Biotin carboxylase	D-serine dehydratase	Internalin A
Glycine betaine uptake system ATP-binding protein YehX	DNA translocase FtsK	Iron-sulfur cluster carrier protein
hypothetical protein	Heat shock protein HslJ	
Multidrug resistance protein MdtB	HTH-type transcriptional regulator CueR	
Multidrug resistance protein MdtC	Multidrug efflux pump subunit AcrB	
Oxaloacetate decarboxylase beta chain	Small toxic polypeptide LdrD	
putative cyclic di-GMP phosphodiesterase PdeC	tRNA (guanosine(18)-2'-O)-methyltransferase	
Ribonuclease G		
Ribosomal protein L11 methyltransferase		
tRNA (cytidine(34)-2'-O)-methyltransferase		
